# Using a behavior change toolkit in pediatric physical therapy to support physical activity: A feasibility study

**DOI:** 10.1371/journal.pone.0286116

**Published:** 2023-11-13

**Authors:** Marleen E. Sol, Elles M. W. Kotte, Eline A. M. Bolster, Sander Hermsen, Remco van der Lugt, Stefan Elbers, Margreet Sanders, Manon A. T. Bloemen

**Affiliations:** 1 Research Group Lifestyle & Health, Knowledge Center for Healthy and Sustainable Living, HU University of Applied Sciences, Utrecht, The Netherlands; 2 Master Education Pediatric Physical Therapy, Institute of Human Movement Studies, HU University of Applied Sciences, Utrecht, The Netherlands; 3 Fitkids Foundation, Amsterdam, The Netherlands; 4 OnePlanet Research Center, Wageningen, The Netherlands; 5 Research Group Co-Design, Research Centre for Learning and Innovation, HU University of Applied Sciences, Utrecht, The Netherlands; University Hospital Cologne: Uniklinik Koln, GERMANY

## Abstract

**Introduction:**

Physical activity levels of children with disabilities are low, as these children and their parents face a wide variety of both personal and environmental barriers. Behavior change techniques support pediatric physical therapists to address these barriers together with parents and children. We developed the What Moves You?! intervention Toolkit (WMY Toolkit) filled with behavioral change tools for use in pediatric physical therapy practice.

**Objective:**

To evaluate the feasibility of using the WMY Toolkit in daily pediatric physical therapy practice.

**Methods:**

We conducted a feasibility study with a qualitative approach using semi-structured interviews with pediatric physical therapists (n = 11). After one day of training, the pediatric physical therapists used the WMY Toolkit for a period of 9 weeks, when facilitating physical activity in children with disabilities. We analyzed the transcripts using an inductive thematic analysis followed by a deductive analysis using a feasibility framework.

**Results:**

For acceptability, pediatric physical therapists found that the toolkit facilitated conversation about physical activity in a creative and playful manner. The working mechanisms identified were in line with the intended working mechanisms during development of the WMY Toolkit, such as focusing on problem solving, self-efficacy and independence. For demand, the pediatric physical therapists mentioned that they were able to use the WMY Toolkit in children with and without disabilities with a broad range of physical activity goals. For implementation, education is important as pediatric physical therapists expressed the need to have sufficient knowledge and to feel confident using the toolkit. For practicality, pediatric physical therapists were positive about the ease of which tools could be adapted for individual children. Some of the design and materials of the toolkit needed attention due to fragility and hygiene.

**Conclusion:**

The WMY Toolkit is a promising and innovative way to integrate behavior change techniques into pediatric physical therapy practice.

## Introduction

Over the last decades, children in the Netherlands follow a worldwide trend of declined physical activity (PA) levels [1–3]. The most recent reports state that only a minority of Dutch children meet national recommendations for overall PA [4]. These recommendations state that childeren, 4–17 years years of age, should participate in 60 minutes or more of moderate-to-vigorous PA each day. This indicates that many Dutch children miss out on the benefits of regular PA, including increased physical fitness, better psychosocial health, and improved health related quality of life [5–8].

When children, with or without a disability, face barriers towards participation in PA, pediatric physical therapists (PPTs) are the designated professionals to support these children because of their core competences in providing prevention and in promotion of health, wellness, and fitness [9]. A PPT treatment plan generally consists of exercise programs, motor learning skills training and/or facilitating sports participation [9, 10]. However, despite these efforts, most interventions do currently not result in long term increased PA [11]. This may be explained by the great variety and complexity of PA determinants within the child’s environmental context [12, 13]. These PA determinants may hinder sustained physical activity, irrespective of the progress during treatment [14].

To generate long-term effects on participation in PA, PPT treatment plans need to change from solely optimizing specific functional components (what is a child able to do) to a broader approach that includes an adequate assessment of personal and environmental determinants associated with participation in PA [11, 15, 16]. Subsequently, there should be an improved focus during treatment on supporting children and their parents to overcome identified barriers to participation in PA. Recent publications recommend incorporating behavior change techniques in PPT treatment programs, for example by including specific tools developed to support people in changing their behavior in the desired direction [11, 17–19]. This shift in pediatric physical therapy follows a trend in (para)medical treatment, where the use of behavior change techniques is integrated in treatment programs to increases the long-term efficacy of health programs [20, 21].

To support PPTs with the integration of behavior change techniques in their treatment plans, we developed an intervention toolkit [14] called the ‘What Moves You?!-Toolkit’ (WMY Toolkit). During the development of the WMY Toolkit, we applied a participatory design approach (co-design) involving designers, developers, researchers, and other stakeholders such as PPTs, parents, children and care sports connectors (CSC) [14]. The design process, involving three co-creation sessions, four one-week design sprints, living-lab testing and two triangulation sessions, led to the development of eleven intervention prototypes; eight physical and three digital tools. By targeting various personal (behavioral/motivational) and environmental components, the tools aim to increase participation in PA in children with physical disability in daily living, such as active transport, playing outside, and sports participation by e.g. stimulating perceived self-efficacy or mapping the child’s physical and social environment. An extensive description of the design proces and the WMY Toolkit is available [14].

To determine to what extend the WMY Toolkit is appropriate for further testing and use, we conducted a feasibility study to provide feedback on the optimization of form and content, and the integration of the intervention in daily clinical practice [22].

## Materials and methods

### Study design

We conducted a feasibility study with a qualitative approach using semi-structured interviews with PPTs as part of the ‘What moves you?!’ project. We provided participating PPTs with a WMY Toolkit prototype and subsequently conducted semi-structured interviews [23] to obtain their experiences with the toolkit in their PPT care setting (primary care or rehabilitation center). We chose semi-structured interviews to gather in-depth information on the experience of PPTs regarding the following four dimensions of feasibility as proposed by Bowen et al. acceptability, demand, implementation, and practicality [22] (see Table 1). The local ethics committee of the University of Applied Sciences Utrecht granted ethical approval for the ‘What moves you?!’ study (99_000_2019).

**Table 1:** Dimensions of feasibility as proposed by Bowen et al. [22].

Acceptability	To what extend is a new idea, program, process or measure judged as suitable, satisfying, or attractive to program deliverers?
Demand	To what extent is a new idea, program, process or measure likely to be used?
Implementation	To what extent can a new idea, program, process, or measure be successfully delivered to intended participants in some defined, but not fully controlled, context
Practicality	To what extend can an idea, program, process, or measure be carried out with intended participants using existing means, resources, and circumstances and without outside intervention?

### Participants and procedure

We recruited PPTs using leaflets spread by the Dutch Association of Pediatric Physical Therapy (NVFK), social media sites such as Facebook and LinkedIn, contacts of researchers from the HU University of Applied Sciences and through *Fitkids*, an exercise therapy program implemented in nearly 165 physical therapy practices in the Netherlands [24, 25]. PPTs were eligible to participate in this study if they treated children with disabilities with PA goals and if they had not participated in the development of the WMY Toolkit.

In December 2019, interested PPTs (n = 50) received a full day of training. The training day consisted of a three-hour interactive lecture and a two-hour practical training session. The aim of the lecture was (a) to explain how to identify barriers and facilitators for effective health behavior change, and (b) to select appropriate behavior change strategies to support the change [26, 27]. The practical training session, which was followed by a selected group of PPTs (n = 24), ensured that therapists had the opportunity to become familiarized with the tools. This session consisted of a demonstration of the tools and an opportunity for the PPTs to use the tools themselves and discuss the potential use. At the end of the practical training session, a selected number of PPTs received a WMY Toolkit (n = 12). This number was equal to the available number of WMY Toolkits at that point. We used a purposive sampling strategy to achieve maximum variation on age, gender and geographical location of the PPT practices. Also work setting (i.e., primary care versus rehabilitation center) was taken into consideration.

We instructed participating PPTs to use the WMY toolkit in at least three children with PA treatment goals during a period of three months (January—April 2020). This included children with and without a disability. Eventhough the WMY Toolkit was developed for use in children with a disability, it became clear from the feedback provided during the design process of the WMY Toolkit, that the WMY Toolkit was possibly also feasible for use in children without a disability. During the inclusion period, PPTs received two email reminders to stimulate usage of the WMY Toolkit. Participants signed a written informed consent for participation in this study and recording of the interview (audio and video).

#### COVID pandemic

In February 2020, the COVID-19 pandemic started in the Netherlands. As a result of this pandemic, face-to-face contact was no longer allowed in PPT practices from March 15^th^ 2020 –May 11^th^ 2020. During that period, therapists were not able to use the WMY Toolkit. This reduced the duration of the intervention period to 9 weeks.

### Material

**Fig 1:**
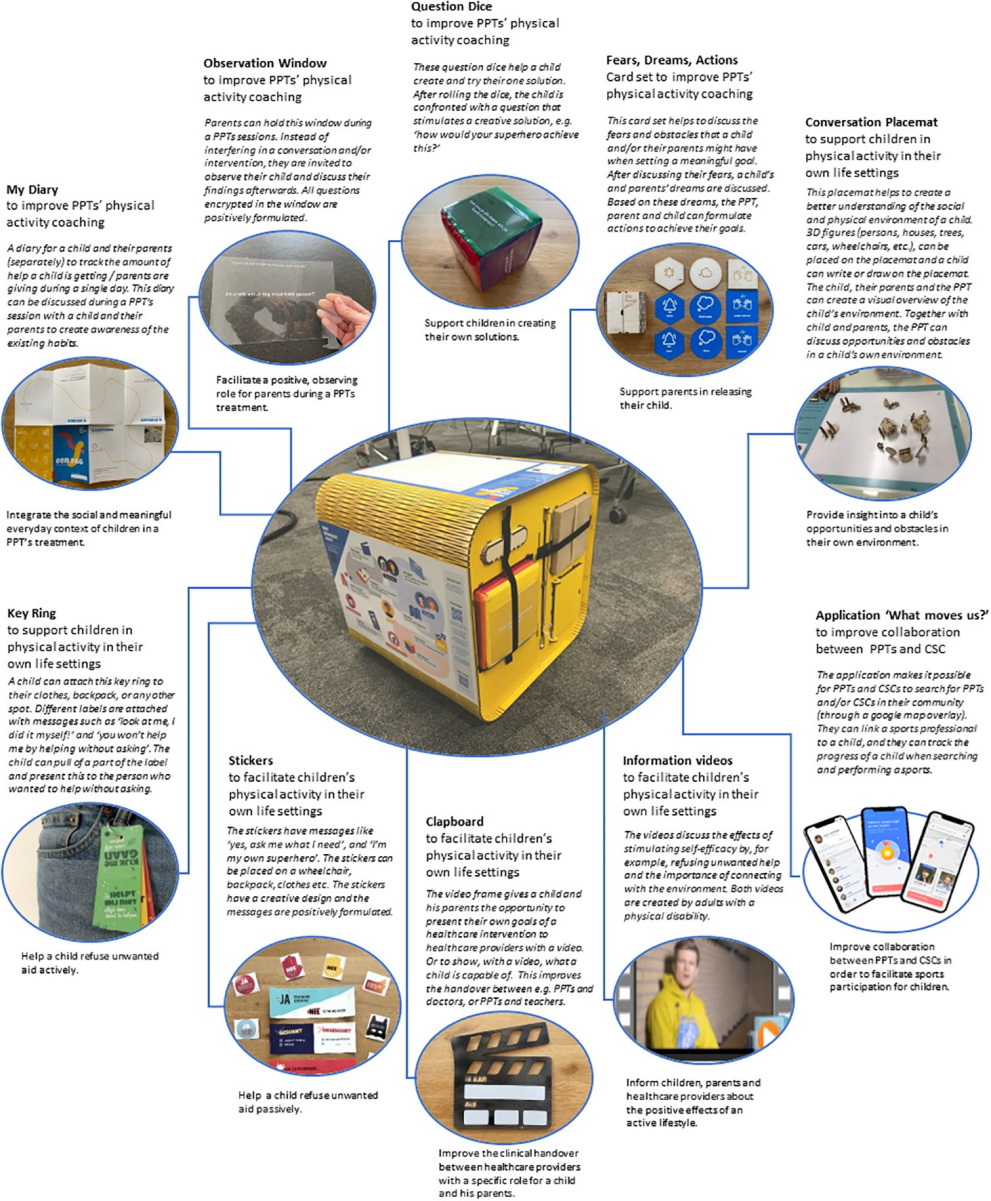
The designed tools including pictures, targeted working mechanisms and description.

The WMY Toolkit consists of eight physical and three digital tools [14]. We developed four physical tools (‘My Diary’, ‘Observation Window’, ‘Question Dice’, ‘Fears Dream Actions Card set’) to improve PPTs physical activity coaching and four physical tools (‘Conversation Placemat’, ‘Key Ring’, ‘Stickers’, ‘Clapboard’) and two information videos to facilitate children’s PA in their daily life settings. A mobile app was developed to improve collaboration between PPTs and CSCs. At the time of this feasibility study, the mobile app needed further development before it was suitable for use in clinical practice. Therefore, it was excluded from the toolkit presented to the participants. The information videos were sent to participating PPTs per email one week after receiving the WMY Toolkit. In this feasibility study, the physical tools were evaluated. The evaluation of the videos was not part of this study. A description of the physical tools is presented in Fig 1. The individual pictured in Fig 1 has provided written informed consent (as outlined in PLOS consent form) to publish their image alongside the manuscript.

### Data collection

A team of three research assistants (MSa, MH, and NP) collected all data (recordings and fieldnotes). The research assistants, all physical therapists, performed this feasibility study as part of their master thesis research. During each interview, MSa was the main interviewer and received support from either MH or NP. The interviews were held online by means of video calling. The research assistants were not professionally related to the participants or their respective treatment centers, nor where they involved in the design process of the WMY Toolkit. They received extensive training by experienced researchers (EB and MB), prior to the interviews. The semi-structured interview guide included questions related to the experiences of the PPTs regarding the acceptability, demand, implementation and practicality of using the WMY Toolkit in daily PPT practice. The research team developed and pretested the interview guide to improve interviewing techniques of the main interviewer.

### Data analysis

We transcribed the interviews verbatim and imported the data into Atlas.ti (version 9; ATLAS.ti Scientific Software Development GmbH) for analysis. First, we used an inductive thematic approach to gather information about the feasibility of using the WMY- toolkit in PPT practice [28]. Two research assistants independently performed ‘open coding’ on all potentially relevant segments. They then sought consensus about selected segments and the coding, if needed involving a third researcher (EB). Thereafter, all ‘open codes’ were inductively organized in subthemes by two research assistants, followed by a consensus meeting with the third researcher. During this process, we organized several critical review sessions (MSa, MH, NP, MS, EB, EK, MB) to enhance credibility and consistency by means of analyst triangulation. Finally, we used the feasibility framework of Bowen et al. to organize all subthemes [22], i.e., acceptability, demand, implementation, and practicality. Consensus regarding this deductive approach was found in two 3-hour research group meetings (MS, EK, MB, EB, MSa). If a subtheme did not fit the framework, an inductive approach was used to determine a possible new theme.

## Results

Eleven PPTs (10 female, 1 male) participated in an individual semi-structured interview, one PPT dropped out due to a high workload related to the COVID-19 restrictions. The interviews lasted between 28 and 70 minutes. The participating PPTs were between 29–56 years of age. Nine PPTs worked in primary care and two in a rehabilitation center. Each therapist used the WMY Toolkit in on average two children (range 1–4) with a median age of 9 years (range 2.5–16 years). The diagnoses of the children were highly heterogeneous, i.e. general motor delay (n = 3), neuromuscular disorder (n = 1), cerebral palsy (n = 4), medically unexplained physical sympotoms (= 1), genetic disorder (n = 1), metabolic disorder (n = 1), sensory processing disorder (n = 1), developmental coordination disorder (n = 2), obesity (n = 2), clubfoot (n = 1), pain (n = 1) and unknown (n = 1). Two children were diagnosed with the comorbidity autismn. In addition, one therapist reported to have used the WMY Toolkit in several children, including clumsy children, without mentioning exact numbers. The children consulted their PPT with a large variety in PA goals, e.g., sports participation, increased physical fitness, improved gross motor skills or increased physical activity.

**Fig 2:**
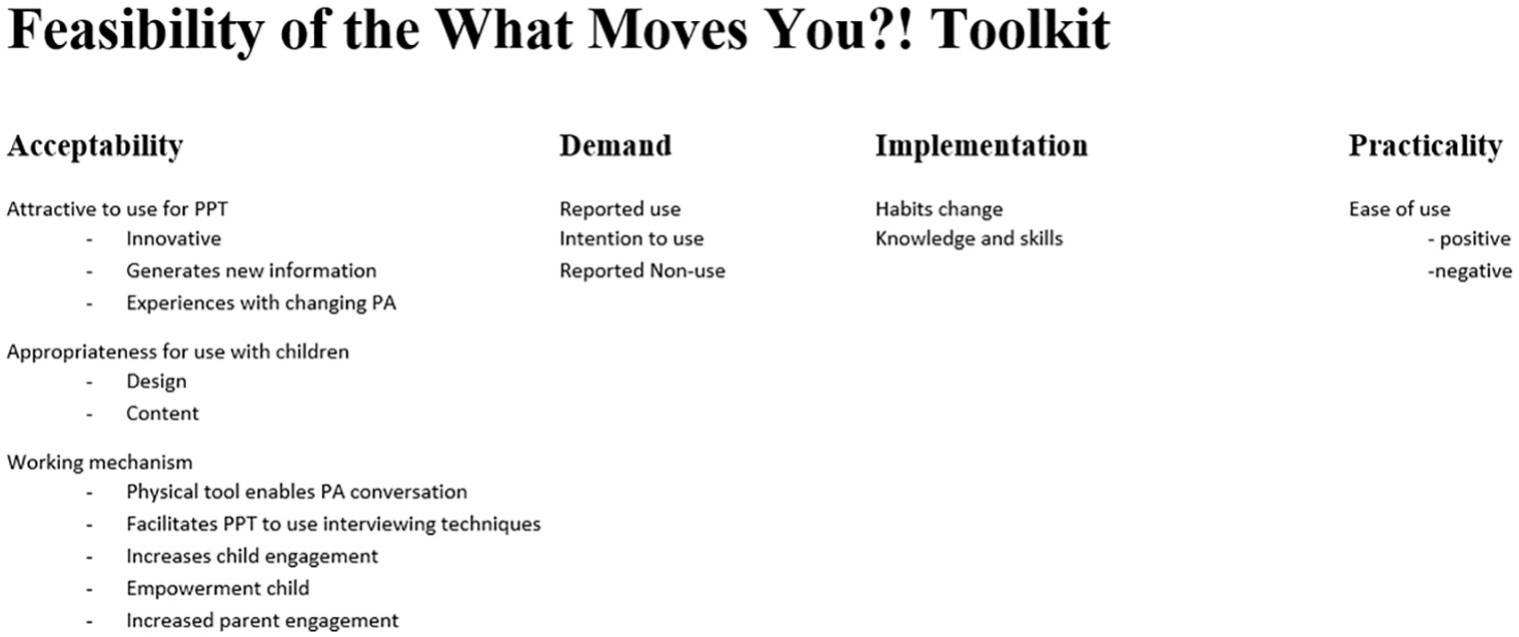
Combined results of the inductive approach (subthemes) and deductive approach (feasibility dimensions).

In Fig 2 we present an overview of the subthemes that were determined during the inductive approach. All subthemes fitted within the predetermined dimensions of feasibility, i.e. acceptability, demand, implementation, and practicality [22], therefore no other main themes were formulated.

### Acceptability

The PPTs experienced the WMY Toolkit as a different method to engage children in the conversation about PA compared to their usual practice (working mechanism). Most PPTs mentioned they enjoyed working with the WMY Toolkit because it was a new method of generating information on PA (attractiveness to use). PPTs expressed that most children found the tools attractive, fun to use and age-appropriate. Some older children (> 12 years of age) found specific features of the tools less age appropriate, e.g. they found the questions on the ‘question dice’ more suitable for younger children (appropriate for youth).

#### Attractive to use for PPTs

All PPTs were positive about using the WMY Toolkit in their daily practice. They felt this toolkit positively added to their current practice as it was a creative and engaging approach for facilitating PA behavior. In addition, several PPTs mentioned they enjoyed the communicational features of the tools, as it was a more creative way to talk about and stimulate PA behavior, compared to their usual PA conservation techniques (question-answer methods, e.g., motivational interviewing techniques).

*PPT #2*: *For me*, *it’s been adding something*. *It really is fun*! *It’s a completely different approach*, *more creative*.

Most PPTs felt that using the toolkit generated new valuable information that could be used for a more detailed description of PA behavior of the child, or which had given them new opportunities for their treatment plan. For example, the information generated could provide insight for PA opportunities in the child’s physical or social environment. Because the information was detailed and concrete, PPTs felt it was easier to help formulate realistic solutions or PA opportunities. Some PPTs mentioned that the toolkit did not always provid new or concrete information, but that it confirmed what they already knew.

*PPT #3*: *For example*, *her girl friends and family were really important to her*. *And so*, *what can we do there*, *in that environment*, *to become more active*.

Another argument for the attractiveness of using the toolkit in PPT practice was the perceived efficacy of the toolkit by some PPTs for positive changes in PA behavior. The PPTs did mention hesitations towards attributing the effect on PA behavior solely to the toolkit, as it was used in addition to their usual care. Most PPTs also commented that they have not (yet) evaluated whether PA behavior actually changed in all children, due to changing circumstances as a result of the COVID-19 pandemic and the short duration of the study period. Nevertheless, most therapists had one or more positive experiences with changing PA behavior in children when using the toolkit.

*PPT #4*: *Uhm*, *I don’t know if this is caused by using the Conversation Placemat*. *But they bought an electric bike*, *and now he uses his bike to go to soccer practice*.

#### Appropriateness for use with children

PPTs reported that the WMY Toolkit appealed to most children. They were positive about the design of most of the tools. They quoted children’s reactions like ‘that’s fun’ or ‘cool’ and commented on the children’s curiosity towards that big special box in the PPT treatment room. Most therapists reported that the playful, less serious approach of the tools was more appropriate for children compared to their usual care of asking (motivational) questions. One therapist did comment that she preferred to talk about PA behavior with a child, while they are already training/practicing and that the design of the tools did not suit that approach. Some therapists commented they were able to adapt the design to suit the child’s age/interest level. They did suggest that it is important for a PPT to know the tool really well before being able to make these adaptations.

*PPT #2*: *I felt the puppets (Conversation Placemat) were too childish for her as she was a bit older (15 years) so we used coloring pencils*, *and just taped that thing to the wall*.
*PPT #5*: *Well*, *for children the dice really is fun*, *because it is different or*…*less serious than just being overflowed with questions*

Some PPTs suggested that the appropriateness of the content of tools depends on a child’s age and cognitive level. One therapist commented on a mismatch between design and content. Whereas the design (dice, drawing, use of imagination) was more suitable for young children, the questions that were part of the tool were abstract and better suited for older children. Some PPTs also shared their doubts about whether the toolkit would be suitable for children with limited cognitive abilities. Many therapists commented that the design of the ‘Observation Window’, where parents had to hold and look through a plastic window, was patronizing. However, they did reported that the actual questions (content) of the ‘Observation Window’ were really good. Overall, PPTs felt that the content of the WMY Toolkit was appropriate for engaging children in talking about PA behavior.

*PPT #6*: *Yeah*, *he was also very enthusiastic*. *He liked it and he also drew a lot*. *He could also use this tool better; I think because he was slightly older (9 years)*.

#### Working mechanisms

The PPTs commented on why they thought the WMY toolkit was different compared to usual care and what they noticed happened in the child or parent when using the WMY Toolkit. Almost all therapists noticed that having a physical tool which children could hold, draw on or see supported them to engage in a PA conversation with the child.

*PPT #7*: *And afterwards I could really start talking with her*. *And so*, *despite her limitations*, *she was able to talk and think about it way better by having something visual*.

Some PPTs also mentioned that they felt the tools facilitated them in using motivational interviewing techniques, e.g. to not give suggestions or solutions, but let the child and parent start answering and find the solution. All PPTs reported that having a physical tool made it easier for the child to talk and think about PA. This enabled children to better express what they wanted to change and how this could be arranged. Almost all PPTs commented that the tools supported them to let the child express the problem or solution in their own words. The PPTs explained how this helped them to understand the perspective of the child and how it facilitated solution orientated thinking in the child.

*PPT #8*: *Because you do it this way*, *the child can think about it longer*, *and I can take step back and be like…*. *I’ll just be quiet now*.

In addition, PPts mentioned noticing that using the tools also lead to another kind of empowerment. For example, PPTs reported that they noticed some children became proud when they followed through with their own solution. PPTs also expressed how it increased awareness and understanding of PA opportunities in a child’s own environment and that some children took more control over their decisions to engage in PA.

*PPT #9*: *Two months ago*, *I used the Conversation Placemat*, *and last week she just mentioned that she walks to school now*. *Even though I hadn’t spoken to her about it at all after using the tool*. *We had used the tool*, *and I did not mention it again*. *But now you see that she did act*.

Some PPTs commented only on what they noticed in the children while others also reported on what they noticed in the parents. The interaction between parents and PPTs differed a lot, depending on the child and/or setting (primary care or rehabilitation center). In primary care, parents were more likely to attend all therapeutic sessions, while in rehabilitation settings PPTs had limited contact with parents and if so, this contact took place mostly by mail or telephone. Overall, most therapists said that the WMY Toolkit supported them in raising awareness in parents about their child’s PA behavior and possible opportunities for increasing PA. For those PPTs who had regular contact with parents, they noticed that some parents had copied the way of phrasing questions to motivate their child at home, such as: ‘What would your superhero do?’ on the ‘Question Dice’. Or that they noticed that parent’s obtained new insights on their child’s perspective on PA opportunities.

*PPT #3*: *She said*, *I see myself in a wheelchair or hand bike*. *And that’s when the parents realized*, *ohhh yeaaah*, *and we just focus on walking*, *walking*, *walking*.

### Demand

#### Reported use

The PPTs reported that they used the WMY Toolkit both with children with and without disabilities, and with a broad range of PA goals such as sports participation and increased PA. All PPTs used the WMY Toolkit during the anamnesis or as an intervention to increase PA. Two PPTs reported that they even used the WMY Toolkit for non-PA related problems, such as writing or sensory impairments to trigger the child to talk about the problems they faced or to become more aware of solutions.

*PPT #10*: *Well*, *I used it with a boy with autismn*, *because they always find it really difficult to tell things*
*by themselves*.

#### Intention to use

Some PPTs expressed situations in which they intended to use the toolkit, but were not able to, due to a variety of circumstances, e.g., therapy focus during intervention period differed from PA problems the child also faced, the high parental burden, or the fact that the WMY Toolkit was less suitable for children that were currently seen in their practice (fine motor skills). All PPTs saw opportunities for using the WMY Toolkit in the future. These opportunities ranged from using it in PA and non-PA related problems in which the tools could help to engage or motivate a child. Some therapists also expressed the opportunities of the WMY Toolkit for other professions such as occupational therapists or social workers.

*PPT #7*: *I also showed it to physical education teachers*, *social workers*, *and pediatric psychologists*. *And they all said*, *what a super good tool and they all saw great potential*.

#### Reported nonuse

Almost all PPTs said that they were highly motivated to start using the WMY Toolkit in daily practice after the training day in December 2019. Before starting, therapists expected they would have numerous potential children in which they could use the WMY Toolkit. Several PPTs expressed that that they had expected to use the tools more often. Reasons for not using the toolbox were, e.g., troubles implementing the WMY Toolkit (see below), the short duration of the intervention period with COVID-19 restrictions and the total number of patients they treated with PA goals. Many PPTs reported that they had a preference towards certain tools, e.g. ‘Question Dice’ and ‘Conversation Placemat’, and had not used others.

*PPT #5*: *and well*, *for a few weeks everybody was thinking*: *Oh*, *I’m gonna use that thing*, *but then it just [smiles] dissappeared in the cupboard*.

### Implementation

#### Habit change

PPTs’ experiences with the ease of implementing the WMY Toolkit in their daily practice varied. One PPT admitted that he/she had forgotten about the WMY Toolkit at some stage because it had disappeared in the cupboard. Some PPTs commented on the fact that courage is needed to use a different method other than usual care. Others felt less comfortable using the WMY Toolkit, as they thought they could not meet the expectations of parents or children who expected physical exercises or ‘doing something’ during PPT sessions instead of ‘talking’. One PPT, commented on this as follows:

*PPT #3*: *It’s just*, *it gave you a lot of ideas*. *But than*, *yeah*, *putting them in practice is something else*.

#### Education

Most PPTs commented on the need of knowing the tools well before being able to decide whether a specific tool is adding something to their usual care. Overall, the training day and additional information helped to motivate PPTs to think about using the WMY Toolkit in daily practice. However, most PPTs said that they needed more experience, to really know how and when it can be used. They needed to feel more confident to really use the WMY Toolkit.

*PPT #7*: *Everybody had to find time for themselves*, *by trying it out*. *And it really isn’t difficult*, *because I think you can just open the toolkit without any preparation*. *And just start experiment with it and use it*.

### Practicality

#### Positive

Overall, PPTs were positive about the practical ease of use of the WMY Toolkit in their practice. They commented that some tools resembled methods they were already familiar with. For example, PPTs were familiar with using a dice, but in the WMY Toolkit the ‘Question Dice’ had questions instead of numbers. Some PPTs were also positive about the ease of which tools could be adapted for individual children, for example, the option of adapting questions in multiple tools. One PPT commented that you did not need any preparation to use the tools.

*PPT #3*: *And well the dice*, *I already use that*. *We made one ourselves with different exercises in it*.

#### Negative

Overall PPTs complained about the fragility of some of the tools, which they understood were related to the fact that the tools were prototypes. Most PPTs gave concrete tips on how to improve the design of the tools or ease of use in daily practice, for example by using plastic materials instead of wood, as plastic is easier to clean. Some PPTs did raise concerns about the time investment for some of the tools during treatment sessions. For instance, they struggled with not having control over the questions and therefore the answers, as the questions were decided by luck of the draw or throw of a dice. Other PPTs reported on difficulties with using the tools with parents, as they struggled to get them engaged due to varying circumstances, e.g. because parents were not always present during PPT sessions. The main complaint was about the size of the WMY Toolkit and one PPT stated:

*PPT #9*: *It’s a huge thing*, *that’s in your way and can’t just put aside somewhere*. *I would use it more*, *if it was easier to bring along*. *It was a bit of a hassle now*.

## Discussion

This study reports on the experiences of PPTs with using the WMY Toolkit in daily PPT practice. The main finding was a positive overall experience regarding the use of the WMY Toolkit, as it was a creative and engaging method for children in talking and thinking about PA. While most of the overall experiences were positive, individual differences in experiences occured per subtheme. For example, whether the WMY Toolkit was appropriate for use in younger or older children, or whether it was easy or difficult to implement the use of the WMY Toolkit in current practice.

There was an extensive report on positive attitudes towards using the WMY Toolkit in daily practice and benefits of using the WMY Toolkit in children, and thus the acceptability of the WMY Toolkit. All PPTs found the WMY Toolkit attractive to use. They described the WMY Toolkit as an innovative tool, which offered a welcome and better alternative to current question-answer methods, e.g., motivational interviewing techniques [29]. PPTs noticed that a physical tool that children could hold on to and play with improved the indepth communication with children. A possible explanation for this success might be found in the field of play therapy, which uses materials such as puppets and toys to communicate with children [30]. Play therapy offers a way for children with low language or abstract thinking abilities to express themselves through playing [31]. While there are only few materials to use in PPT practice that might support conversations on participation in PA, the WMY Toolkit is a promising new intervention to use in PPT practice [14]. As in play therapy, children are encouraged to express themselves in a nondirective, interactive, and fun way that corresponds with the child’s world.

Another interesting result regarding the acceptability of using the WMY Toolkit were the explanations PPTs gave about how they experienced the working mechanism of the WMY Toolkit (see Box 1. Case example). For example by empowering the child, by increasing child engagement, or by stimulating conversations between the child and parents on PA opportunities. Most of these working mechanisms are in line with those described in the design study of the WMY Toolkit [14] and in line with behavior change technique [20]. The satisfaction of the PPTs with noticing behavior change through the use of the WMY Toolkit strengthens the acceptability of using the toolkit in PPT settings.

**Case example**

This is a description of how a pediatric physical therapist, who works at a special needs school, used the WMY-Toolkit with a 11-year-old girl “Sophie”. Sophie is diagnosed with spastic bilateral cerebral palsy (GMFCS II) and a mild mental retardation. Sophie walks in and around school and uses a wheelchair at home for longer distances. She wants to participate in sports and asked her PPT to help her.

Her PPT chose to use the ‘Conversation Placemat’ to get a clear overview of when and how (walking or using her wheelchair) she is physically active during and after schooltime. By using drawings and walking/wheelchair puppets a scene appeared where she used the wheelchair more often in situations at home than at school. This started a conversation between Sophie and her PPT about current physical activity behavior.

*PPT #1*: *“Despite her limitations*, *she could make it visual*, *which made it a lot easier for her to think and talk about it”*

Without adressing any areas for change in being physically active, Sophie mentioned in the next treatment session that she walked to a family visit instead of using her wheelchair. The PPT was impressed that the tool had already encouraged a change in PA behavioral without talking over any solutions with Sophie.

*PPT #1*: *“And then*, *in the spring holiday*, *she convinced her parents that she could go to a try-out training session at a sportclub*. *She took the initiative to do that*, *while she would never have done that before*. *Something had really changed*!*”*

Not all experiences regarding the use of the WMY Toolkit were positive. While all PPTs claimed they often treated children with PA goals and were highly motivated to start using the WMY Toolkit, almost all PPTs were disappointed to report that they could not use the toolkit more frequently during the study period. A possible explanation for this was given by PPTs themselves: they noticed that parents often contact a PPT for their child regarding a functional problem rather than a request to increase participation in PA in their children. While PPTs often suspected limited participation in PA, they may have chosen to only address the functional problem presented. This could explain the difference between the number of children with limited participation in PA that seek help from a PPT and how often treatment goals actually are aimed at increasing participation in PA. Future research should investigate how PPTs might more easily focus on the level of participation rather than on the level of bodily functioning as defined by the ICF [32]. Another possible explanation for the limited use of the WMY Toolkit might come from barriers to behavior change within PPTs themselves. As a strategy to start using the WMY Toolkit, PPTs only received one day of training. PPTs expressed they needed more experience and wanted to feel more confident to start using the WMY Toolkit. Integrating behavior change techniques within PPT practice is a truly different approach compared to usual care in which physical exercises are more prominent [33]. This requires different competencies (e.g., coaching skills to stimulate self-efficacy and autonomy of the child), takes time and more training than just one day. Furthermore, from the perspective of behavior change theories and implementation science, the implementation of new interventions needs more attention than just an educational strategy but should also focus on facilitating PPTs on how to adapt their methods to include this new WMY Toolkit [20, 34].

During the design process of the WMY-Toolkit, co-creation sessions had already resulted in extensive information on individual tools [14]. However, there was only limited information available on using the toolkit as a complete set in PPT practice. Therefore, the focus of this feasibility study was to assess the use of WMY Toolkit as a complete set rather than to evaluate the feasibility of separate tools. Nevertheless, the ‘Conversation Placemat’ and the ‘Question Dice’ were most frequently used by PPTs, possibly indicating that these tools are more feasible or more comparable to current PPT practice. Future research is needed to evaluate feasibility of the individual tools in the WMY Toolkit. Another limitation of the study design was the collection of experiences only from the perspective of PPTs. In the original project we also aimed at including perspectives of children and parents. Unfortunately, data collection took place during the start of the COVID-19 pandemic and many parents and children were unavailable due to the stress of finding a new balance between work, home schooling, and being at home under the COVID-19 restrictions. This made it impossible to include parental and child perspectives in the current study. For a broader overview of the feasibility of the WMY Toolkit, future studies should include the perspective of children and their parents. Furthermore, the COVID-19 restrictions had impact on the ability to use the WMY Toolkit, especially during the last three weeks of the study, as PPT locations were then closed. Despite these limitations, we were able to report on a broad range of experiences from PPTs using the WMY Toolkit in daily practice. A major strength of this study was that participating PPTs worked in different settings (primary care and rehabilitation centers) and in different geographical locations in the Netherlands, which led to an assessment of feasibility of the WMY Toolkit in a highly heterogeneous group of PPTs. Several PPTs commented that they showed the WMY Toolkit to their colleagues (physical education teachers, social workers, and teachers) or even used some of the tools in not PA-related therapeutic questions such as writing. Future research should address the question whether the WMY Toolkit can be adopted to different populations and professions [22]. Finally, this study was a feasibility study to analyze experiences of PPTs in using the WMY Toolkit in daily practice. The results of this study will be used to further develop de WMY Toolkit for use in PPT practice and assess the efficacy of the WMY Toolkit in future studies.

## Conclusion

The WMY Toolkit is a promising and innovative way to integrate behavior change techniques into PPT practice. PPTs experienced that the tools facilitated conversation about PA in children and their parents in a creative and playful manner. They shared their thoughts regarding the working mechanisms of the WMY Toolkit, which was in line with the intended working mechanisms during development, such as focusing on possibilities rather than on obstables, stimulating self-efficacy and stimulating autonomy. Using the WMY Toolkit, and thus integrating behavior change techniques into PPT practice, requires behavior change of PPTs themselves. Appropriate training and implementation seems to be essential to change current PPT practice. The efficacy of the WMY Toolkit should be analyzed in an additional study.
